# Alzheimer's disease plasma biomarkers are differentially affected by Type II Diabetes Mellitus based on those with Alzheimer's disease

**DOI:** 10.1002/alz70856_106863

**Published:** 2026-01-11

**Authors:** John Grizzanti, Xuemei Zeng, Rebecca A Deek, Michel N Nafash, Jeremy M. Gu, Lamia Choity, Tara K Lafferty, Marissa F Farinas, Margaret A Bedison, Rocco B Mercurio, Cristy Matan, Alexandra Gogola, Julia K. Kofler, Dana L Tudorascu, C. Elizabeth Shaaban, Jennifer H Lingler, Tharick A Pascoal, William E Klunk, Victor L. Villemagne, Milos D. Ikonomovic, Sarah B Berman, Robert Sweet, Beth E. Snitz, Ann D Cohen, M. Ilyas Kamboh, Oscar L Lopez, Thomas K Karikari

**Affiliations:** ^1^ University of Pittsburgh Medical School, Pittsburgh, PA, USA; ^2^ University of Pittsburgh School of Medicine, Pittsburgh, PA, USA; ^3^ University of Pittsburgh, Pittsburgh, PA, USA; ^4^ School of Public Health, University of Pittsburgh, Pittsburgh, PA, USA; ^5^ University of Pittsburgh Alzheimer's Disease Research Center (ADRC), Pittsburgh, PA, USA; ^6^ University of Pittsburgh, School of Medicine, Pittsburgh, PA, USA; ^7^ The University of Pittsburgh, Pittsburgh, PA, USA

## Abstract

**Background:**

Type II Diabetes Mellitus (T2DM) remains one of the strongest comorbidities associated with the development of AD. Use of fluid‐based biomarker assays allows for quick, inexpensive, and accurate identification of those at high risk for developing AD or likely already have the disease. While AD and related dementias (ADRD) fluid biomarkers are well characterized during aging and cognitive impairment, little is known about the effects of T2DM on ADRD fluid biomarkers, especially those related to tau.

**Method:**

Participants with relevant demographic, diagnostic, and ADRD plasma biomarker data from the University of Pittsburgh's Alzheimer's disease Research Center were utilized for this study (*N* = 1,140). Participants were stratified by cognitive diagnosis [CogDx] (Cognitively unimpaired [CU], MCI, or AD) and diabetes (non‐diabetic [ND] or T2DM). ADRD plasma biomarkers were analyzed using single molecule array (Simoa): Brain‐derived (BD) tau, *p*‐tau181, *p*‐tau217, NfL, and GFAP. After a log2‐transformation, ADRD plasma biomarkers were analyzed via 2‐way ANCOVA with a Bonferroni correction while controlling for age.

**Result:**

Of the 1,140 included participants 150 (13.2%) were diagnosed with T2DM. Most participants were classified as AD (523, 45.9%) or MCI (305, 26.8%). There was no mean age difference between participants ± T2DM. There was a stepwise difference in age between CU<MCI<AD participants (*p* <0.001). 2‐way ANCOVA analysis of plasma biomarkers: *p*‐tau181, BDtau, NfL, and GFAP all increased stepwise from CU<MCI<AD, (*p* <0.001). GFAP (*p* <0.001) and NfL (*p* = 0.036) levels were also separately affected by diabetes and were lower in those with T2DM compared to NDs. Lastly, *p*‐tau217 showed an interaction between CogDx and T2DM (*p* = 0.05), with a stepwise increase from CU to MCI to AD in ND, but similar levels between T2DM+CU and T2DM+MCI and a higher level in T2DM+AD than both groups.

**Conclusion:**

All ADRD plasma biomarkers showed a clear, stepwise increase based on CogDx (CU<MCI<AD), recapitulating previous works. Novel data demonstrated an unanticipated decrease in plasma GFAP and NfL in those with T2DM. Lastly, *p*‐tau217 was preferentially increased in participants with AD+T2DM vs. T2DM participants that were CU or with MCI. These novel findings require further scrutiny within this cohort and others.

